# Structural and Functional Analysis of SsaV Cytoplasmic Domain and Variable Linker States in the Context of the InvA-SsaV Chimeric Protein

**DOI:** 10.1128/Spectrum.01251-21

**Published:** 2021-12-01

**Authors:** Jinghua Xu, Jiuqing Wang, Aijun Liu, Yanqing Zhang, Xiang Gao

**Affiliations:** a State Key Laboratory of Microbial Technology, Shandong University, Qingdao, China; b Shanghai Fifth People's Hospital and Shanghai Key Laboratory of Medical Epigenetics, International Co-laboratory of Medical Epigenetics and Metabolism (Ministry of Science and Technology), Institutes of Biomedical Sciences, Fudan University, Shanghai, China; c School of Life Sciences, Shandong University, Qingdao, China; University of Guelph

**Keywords:** *Salmonella*, type III secretion system, injectisome, export gate protein, cryo-EM

## Abstract

The type III secretion (T3S) injectisome is a syringe-like protein-delivery nanomachine widely utilized by Gram-negative bacteria. It can deliver effector proteins directly from bacteria into eukaryotic host cells, which is crucial for the bacterial–host interaction. Intracellular pathogen Salmonella enterica serovar Typhimurium encodes two sets of T3S injectisomes from Salmonella pathogenicity islands 1 and 2 (SPI-1 and SPI-2), which are critical for its host invasion and intracellular survival, respectively. The inner membrane export gate protein, SctV (InvA in SPI-1 and SsaV in SPI-2), is the largest component of the injectisome and is essential for assembly and function of T3SS. Here, we report the 2.11 Å cryo-EM structure of the SsaV cytoplasmic domain (SsaV_C_) in the context of a full-length SctV chimera consisting of the transmembrane region of InvA, the linker of SsaV (SsaV_L_) and SsaV_C_. The structural analysis shows that SsaV_C_ exists in a semi-open state and SsaV_L_ exhibits two major orientations, implying a highly dynamic process of SsaV for the substrate selection and secretion in a full-length context. A biochemical assay indicates that SsaV_L_ plays an essential role in maintaining the nonameric state of SsaV. This study offers near atomic-level insights into how SsaV_C_ and SsaV_L_ facilitate the assembly and function of SsaV and may lead to the development of potential anti-virulence therapeutics against T3SS-mediated bacterial infection.

**IMPORTANCE** Type III secretion system (T3SS) is a multicomponent nanomachine and a critical virulence factor for a wide range of Gram-negative bacterial pathogens. It can deliver numbers of effectors into the host cell to facilitate the bacterial host infection. Export gate protein SctV, as one of the engines of T3SS, is at the center of T3SS assembly and function. In this study, we show the high-resolution atomic structure of the cytosolic domain of SctV in the nonameric state with variable linker conformations. Our first observation of conformational changes of the linker region of SctV and the semi-open state of the cytosolic domain of SctV in the full-length context further support that the substrate selection and secretion process of SctV is highly dynamic. These findings have important implications for the development of therapeutic strategies targeting SctV to combat T3SS-mediated bacterial infection.

## INTRODUCTION

The type III secretion system (T3SS) is a supramolecular nanomachine employed by numerous Gram-negative bacteria to facilitate pathogenic or symbiotic interactions between microbes and their eukaryotic hosts (1, 2). The T3SS, an ∼3.5 MDa protein complex consisting of more than 20 proteins, possesses two types of bacterial molecular machines, the flagellum and injectisome, with highly conserved architectures but different functions (3–5). The flagellum is a crucial apparatus involved in bacterial motility (6). The injectisome sits in the bacterial envelope and spans three biological membranes: the inner and outer membranes of the bacteria, and the membrane of host cells. This structure thus bridges individuals from two kingdoms with a hollow conduit, through which bacterial effectors can be directly delivered into eukaryotic host cells and modulate hosts’ physiological functions (1, 7–9). Type III secretion (T3S) injectisome comprises several protein complexes, including the sorting platform, export apparatus, basal body, needle, and translocon (10–12). Each complex is made up of one or more proteins. The well-organized assembly and effective cooperation of these complexes ensure that the T3S injectisome can inject effectors from bacteria into the host cells with precise regulation (12–14).

**FIG 1:**
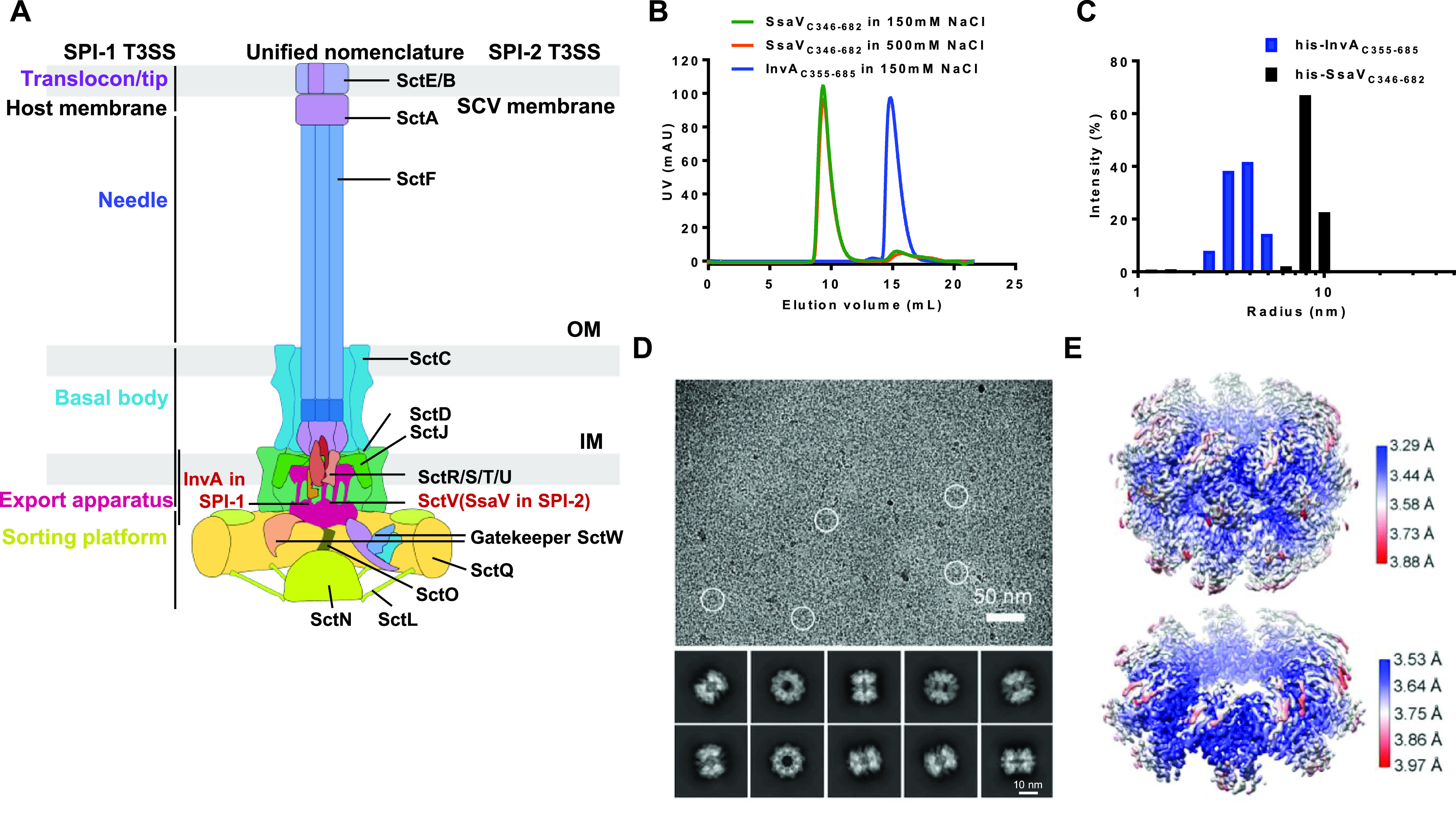
SsaV_C_ tends to be a stable nonamer compared with InvA_C_ in solution. (A) Cartoon representation of T3SS injectosome in SPI-1 and SPI-2. SctV is shown in red. (B) Gel filtration traces of purified InvA_C_ and SsaV_C_ in two different salt concentrations. InvA_C_ elutes as a monomer while SsaV_C_ elutes as a nonamer under physiological and high salt conditions. (C) Oligomeric states of SsaV_C_ and InvA_C_ measured by Dynamic Light Scattering (DLS). (D) Representative micrograph and 2D class averages of SsaV_C_. Representative particles in the micrograph are highlighted with white circles (top panel). Double layer features of the SsaV_C_ are obvious in the 2D class averages (bottom panel). (E) Local resolution map of SsaV_C_ with D9 symmetry with an average resolution at 3.55 Å resolution (top panel) and C9 symmetry with an average resolution at 3.64 Å resolution (bottom panel). The local resolution maps were presented with UCSF Chimera.

The intracellular bacterial human pathogen Salmonella enterica serovar Typhimurium possesses two sets of T3S injectisome, which are encoded in Salmonella pathogenicity islands 1 and 2 (SPI-1 and SPI-2) (15, 16). SPI-1 T3SS is activated and assembled in extracellular bacteria and primarily facilitates the invasion of host cells (17, 18). After entry into host cells, the bacteria form a Salmonella-containing vacuole (SCV), then utilize the SPI-2 T3SS to mediate host cell signaling in order to promote its own growth, replication, and further dissemination in host tissues (19–23). Although the functions of effectors secreted by SPI-1 and SPI-2 T3SSs are largely different, most of the building blocks are highly conserved between the two systems, both in structure and function (Fig. 1A).

The export gate protein SctV (InvA in SPI-1 and SsaV in SPI-2), located at the outermost layer of the export apparatus, is the largest and the first identified component of the Salmonella T3S injectisome (12, 24). SctV comprises a highly conserved N-terminal transmembrane (TM) domain and a less conserved C-terminal cytoplasmic domain (SctV_C_), which are connected via a ∼20–40 amino acid linker, SctV_L_, in most bacteria using the T3SS (12). It has been reported that SctV is one of the “engines” of T3SS (25); the TM domain forms a putative proton channel, and SctV_C_ forms a nonameric ring connecting to SctN (ATPase) through SctO (the stalk protein) to function as the F_0_F_1_-ATPase, coupling energy from ATP hydrolysis and the proton-motive force to secrete unfolded bacterial effectors into the eukaryotic host (26–29). SctV_C_ is also involved in substrate selection through recognizing gatekeeper proteins or different effector-chaperone pairs (15, 30–34). A previous study showed that substituting homologous TM and cytoplasmic domains between some SctV proteins caused them to retain their functions, but that SctV_C_ controls substrate specificity (35).

Intensive structural and functional studies of T3SS have uncovered much detailed structural information and potential assembly processes of this complicated molecular machine (12, 36–40). However, the structure and molecular mechanism of SctV are mostly unknown due to the challenges of obtaining the fully assembled state of the full-length protein. Several studies have isolated the entire T3S injectisome and flagellar basal body for structural studies (12, 41–43). However, SctV was missing in all these trials, even though all other export apparatus components could be captured; this suggests a loose interaction between SctV and other components of the export apparatus and the basal body. The structure of SctV_C_ has been identified as a homo-nonamer through crystal and cryo-electron microscopy (cryo-EM) structural studies, and the intermolecular polar interactions between monomers are thought to be the leading force maintaining the SctV_C_ oligomeric state (26, 44–46). Recently, an *in situ* cryo-electron tomography (cryo-ET) study identified the location of InvA in the bacterial inner membrane and showed a high-order oligomeric state of the TM domain of InvA (12). More recently, Matthews-Palmer et al. and Kuhlen et al. obtained assembled full-length SctV and FlhA suitable for cryo-EM structural studies (47, 48). However, due to the structural flexibility issues, both groups only determined the structure of SctV_C_ and FlhA_C_, leaving the structure of linker region and the TM domain still unknown.

In this study, through generating a chimeric protein consisting of the TM region of InvA and the cytoplasmic region of SsaV, we produced the full-length SctV in a high-order oligomeric state which was adequate for structural study using cryo-EM single particle analysis. Here, we present the 2.11 Å nonameric ring structure of SsaV_C_ with a semi-open state of each monomer. Moreover, we display the cryo-EM structure of SsaV_L_ through the single particle analysis for the first time although with low resolution. Interestingly, SsaV_L_ exhibits two major orientations, consistent with previous reports that the conformations of the SctV linker region could be altered during the secretion cycle (49, 50). Structural analysis and biochemical assays indicate that SsaV_L_ plays an essential role in maintaining the nonameric state of SsaV. Collectively, our data provide an atomic view and mechanistic understanding of how the cytoplasmic domain and linker region of SctV facilitate its assembly and function.

## RESULTS

### SsaV_C_ forms a stable homo-nonameric ring.

To determine the structure of SctV, we initially tried to purify the full-length InvA protein from Salmonella SPI-1 T3SS. However, we were unable to obtain a stably-assembled InvA sample for the cryo-EM structural study, even after several rounds of high-throughput detergent screening. It has been shown that SctV_C_ makes a significant contribution in maintaining the SctV nonamer (46). Previous structural studies suggested that InvA_C_ tends to be a monomer in solution (51). Therefore, it may be challenging for the full-length InvA to form the stable nonamer outside of the membrane. To obtain a fully assembled SctV sample, we first tested the nonamerization ability of SsaV_C_, the homologous protein of InvA_C_ from Salmonella SPI-2 T3SS. Unlike InvA_C_, SsaV_C_ could maintain a very stable high oligomeric state even in the high salt concentration analyzed by the size exclusion chromatography (SEC; Fig. 1B) and dynamic light scattering (DLS; Fig. 1C). Through a cryo-EM single particle analysis approach, we reconstructed and classified two conformations of SsaV_C_: one 3.55 Å cryo-EM structure with double stacked nonameric rings with D9 symmetry, and the other 3.64 Å cryo-EM structure with a single nonameric ring with C9 symmetry, the particle number of which are approximately comparable (Fig. 1D and E and Fig. S1 in the supplemental material). The double-layer ring conformation of SsaV_C_ is considered to be an artifact, consistent with a previous study (45). Notably, most of the C9 single-layer ring of the SsaV_C_ particles were calculated from top views, and most of the D9 double-layer ring of SsaV_C_ particles came from side views. We speculate that this is because the top-view protein particles are more vulnerable to air–water interface damage than the side-view particles (52). Together, unlike InvA_C_, SsaV_C_ could form the stable homo-nonameric ring in solution.

### InvA_TM_-SsaV_C_ chimeric full-length protein can assemble into a nonamer.

**FIG 2:**
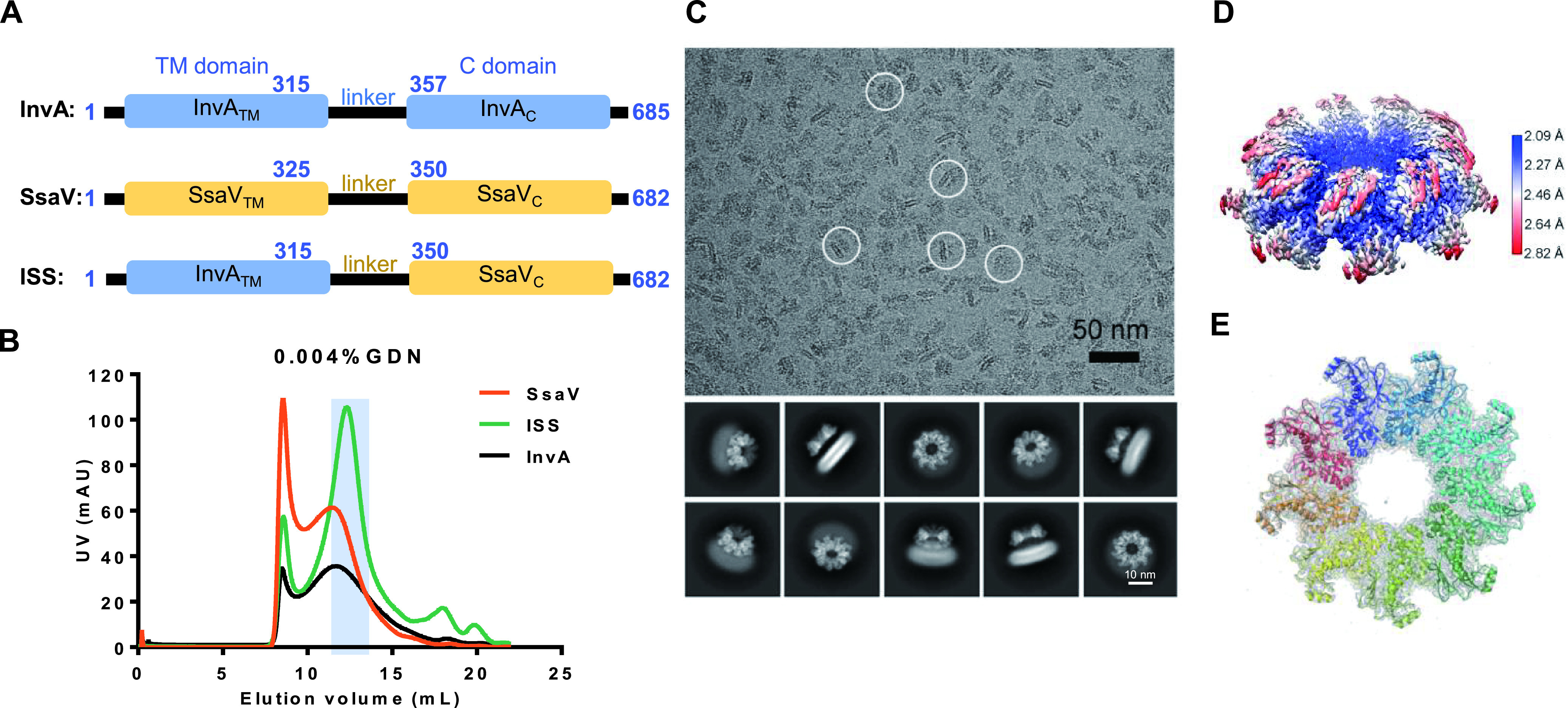
InvA_TM_-SsaV_C_ chimera (ISS) forms a nonamer in GDN micelles. (A) A diagram for the chimera design. Residues 316–685 of InvA are replaced by residues 326–682 of SsaV. (B) Final SEC purification of the chimeric ISS, InvA and SsaV full-length protein in 0.004% GDN. The main peak fractions of the chimeric ISS (highlighted in the light blue background) are used for cryo-EM analysis. (C) Representative micrograph and 2D class averages of the chimeric ISS. Characteristic particles in distinct orientations are highlighted by white circles (top panel). TM region of the chimeric ISS is visible clearly in 2D class averages (bottom panel). (D) The local resolution map of the chimeric ISS is at 2.11 Å resolution, while the high-resolution part is SsaV_C_. The TM and linker regions are hardly visible at this resolution because of structural flexibility. The local resolution maps were presented with UCSF Chimera. (E) The atomic model of SsaV_C_ in rainbow fitted into its EM-map in gray.

To test if the full-length SsaV and the TM domain of InvA could form the stable nonamer in solution with SsaV_C_, we constructed the full-length SsaV protein and the chimeric full-length protein InvA_TM_-SsaV_L_-SsaV_C_ (ISS; Fig. 2A). We performed a high-throughput detergent screening to identify suitable detergents to help stabilize the assembled state of SsaV and ISS. However, very few detergents could generate protein samples of high enough quality for cryo-EM structural study. A homogeneous and oligomerized ISS protein was eventually obtained in buffer containing the detergent Glyco-diosgenin (GDN; Fig. 2B); however, SsaV (similarly to InvA) could not form the stable nonamer in this condition (Fig. 2B). Compared with full-length SsaV and InvA, GDN-solubilized ISS protein showed better SEC and cryo-EM micrograph behaviors (Fig. 2B and C) and were suitable for cryo-EM data processing.

Further 2D classification and 3D reconstruction of ISS showed the apparent density of the SsaV_C_ nonameric ring, which is consistent with structural features of the SsaV_C_ domain described above (Fig. 2C and D and Fig. S2). We reconstructed a high-resolution cryo-EM structure of SsaV_C_ with an averaged resolution of 2.11 Å (Fig. 2D and Fig. S2). Unlike SsaV_C_, for which we obtained high-resolution structural information, the EM density of InvA_TM_ is blurry, and the SsaV_L_ EM density is absent, with an approximately constant distance between the cytoplasmic domain and TM domain; this is consistent with a recent study (47). In summary, we found that the GDN-solubilized InvA_TM_-SsaV_C_ chimeric full-length protein tended to assemble into a nonamer in solution.

### Structure of SsaV_C_.

**FIG 3:**
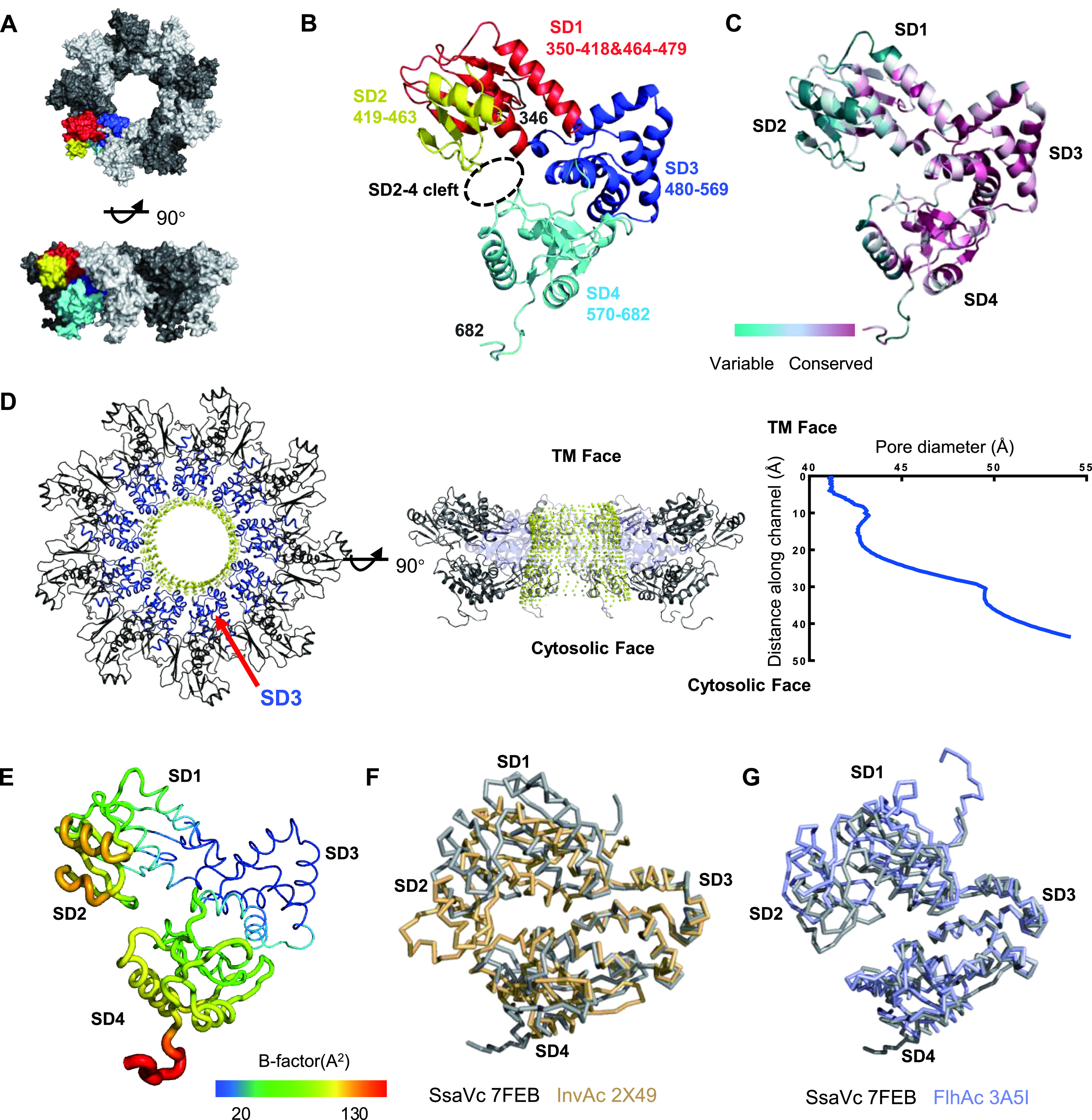
Structure analysis of SsaV_C_. (A) Surface representation of SsaV_C_ nonamer, top and side views. The colored monomer corresponds to SsaV_C_ monomer. (B) The SsaV_C_ monomer with SD1 (residues 350–418, 464-479) in red; SD2 (residues 419–463) in yellow; SD3 (residues 480–569) in blue; SD4 (residues 570–682) in cyan. The dotted circle in black represents a cleft between SD2 and SD4. (C) Sequence conservation of SsaV_C_ colored according to residue identity conservation scores obtained by ConSurf. These amino acid sequences are from 16 members of the SctV family. (D) Diameters of the channels at the center of SsaV_C_ nonamer. The channels illustrated by yellow dots are mainly surrounded by SD3 shown in blue in top view (left panel). Side view of the channels in SsaV_C_ nonamer (middle panel). The pore diameter diagram calculated using the Hole program in Coot (right panel). (E) Cartoon representation of SsaV_C_ monomer colored according to B-factor values. (F-G) Overlay of SD3 of SsaV_C_ monomer in gray with InvA_C_ in a closed state (wheat, PDB:2X49) and FlhA_C_ in an open state (purple, PDB:3A5I). The SsaV_C_ monomer is shown in a semi-open conformation.

Based on the 2.11 Å cryo-EM map of ISS solved above (Fig. 2D and Fig. S2), we built an atomic model of the SsaV_C_ nonamer by referring to the SsaV_C_ monomer structure (PDB: 7AWA) solved recently (47) (Fig. 2E, Fig. 3A, Fig. S3, and Table S1 in the supplemental material). The high structural similarity between these two SsaV_C_ monomer structures (Cα RMSDs: 1.3598 Å) indicates that they hold a consistent structural conformation in the context of different TM regions (Fig. S4). Consistent with other reported SctV_C_ structures (26, 44, 45, 47, 50, 53), the SsaV_C_ monomer also has a four-subdomain (SD) structure and further assembles to a nonameric ring aligned to SD3, which is the most conserved region of SsaV_C_ (Fig. 3A to D). The diameter of the channel at the center of the SsaV_C_ nonamer ranges from ∼54-41 Å from the cytosolic face to the TM face (Fig. 3D). This channel further connects to the channel formed by other components of the export apparatus (SctRST) and finally extends to the needle conduit with a diameter of ∼25 Å (54). Through this reverse funnel-like channel, T3SS systematically unfolds effector proteins and secretes them out of the bacteria. During the effector secretion process, SsaV_C_ was reported to involve substrate selection with the cleft between SD2 and SD4 (13, 32). The dynamic conformations between SD2 and SD4 have been demonstrated through both different SsaV_C_ structures and molecular dynamic simulation, showing that SD2 and SD4 can alternate between open and closed states hinging around the rigid SD3 (13, 26, 44, 45, 47).

Consistent with the observation above, B-factor analysis of the SsaV_C_ structure also shows that SD3 is the most stable region of SsaV_C_ with the more flexible SD2 and SD4 floating around it (Fig. 3E). Structural comparison between SsaV_C_ and InvA_C_ (51) in a closed conformation or its counterpart in flagellum, FlhA_C_ (55), in an open conformation shows that SsaV_C_ in a full-length context presents a semi-open conformation (Fig. 3F and G). Recent structural study revealed that the YscV_C_ (the homolog of SsaV from Yersinia enterocolitica) and FlhA_C_ in a full-length context present in the open state (48). Comparison of our SsaV_C_ structure with these two reported homologous structures also exhibits the different conformations between SD2 and SD4 (Fig. S5 in the supplemental material). Together, these findings firstly show the dynamic conformations between SD2 and SD4 of SctV in a full-length context.

### The essential roles of linker region for the structural stability and function of SsaV.

**FIG 4:**
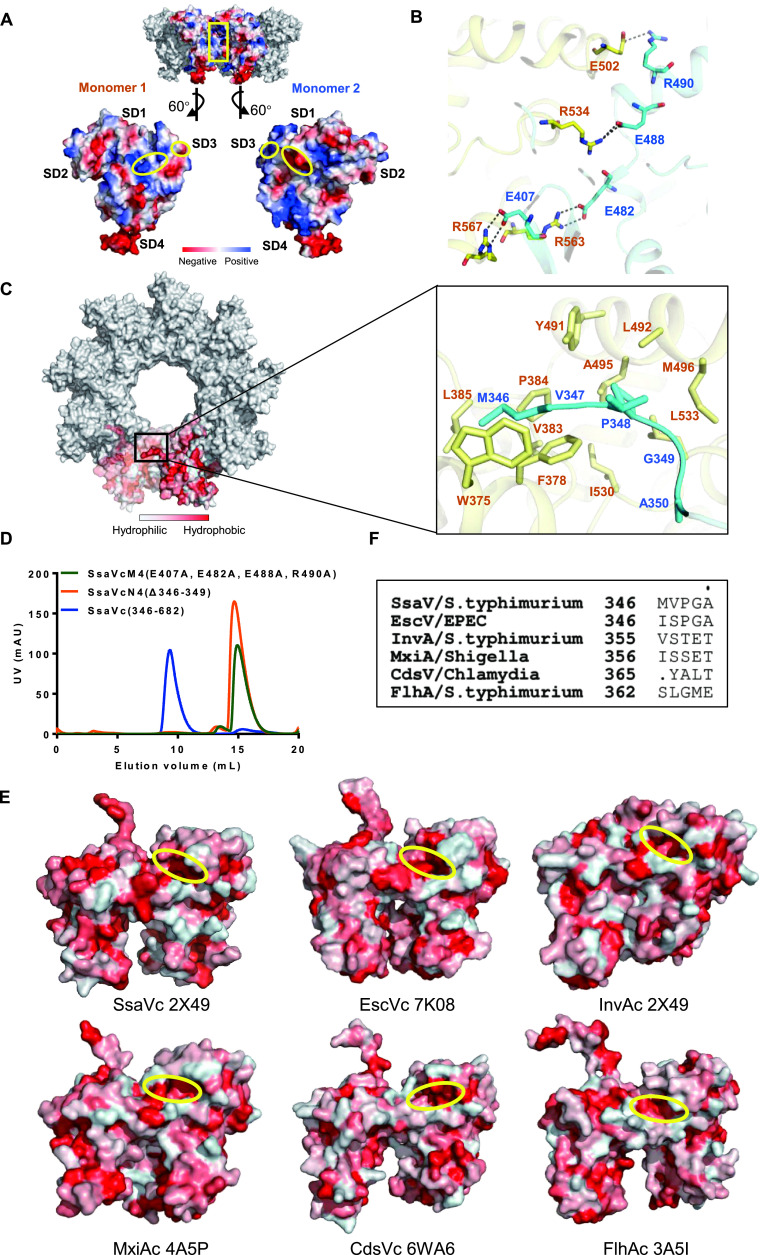
The linker region is important for assembly of the SsaV_C_ nonameric ring. (A) The electrostatic surface between two adjacent monomers of SsaV_C_. The complementary electrostatic interactions in SD1-SD3 are highlighted by yellow circles. (B) Cartoon of the interface between two adjacent monomers, showing intermolecular salt-bridges residues as sticks: E407-R567, R563-E482, R534-E488, E502-R490. (C) Analysis of hydrophobic interaction between two adjacent monomers. The cartoon presentation of the hydrophobic interactions is shown enlarged in the right panel. A hydrophobic peptide (346-350) shown as sticks from the linker region (cyan) is nestled into a hydrophobic pocket in the neighboring monomer (yellow). These residues in the hydrophobic pocket with their side chains are shown as sticks. (D) Gel filtration traces of purified SsaV_C_, SsaV_C_N4(Δ346-349), and SsaV_C_M4(E407A, E482A, E488A, R490A). SsaV_C_N4, with a deletion of the hydrophobic peptide (residues 346–349) and SsaV_C_M4, with mutations in the salt-bridges, cannot form a nonameric ring. (E) Surface hydrophobic analysis in different homologous proteins. The similar locations of the hydrophobic pockets described in C are highlighted with yellow circles in these proteins. (F) Sequence alignment of the hydrophobic peptides from the linker region in different homologous proteins with solved structures. These residues appear to have different properties, with the hydrophobicity in SsaV stronger than in others.

To gain insights into the molecular mechanism of SsaV_C_ nonamerization, we analyzed the electrostatic surface potential of the interfaces between SsaV_C_ monomers within the nonamer and found that electrostatic interactions in SD3 and SD1 of SsaV_C_ may facilitate the subunit nonamerization (Fig. 4A). Close inspection of the oligomerization interfaces revealed that four pairs of salt bridges presenting in SD3-SD3 (R534-E488, E502-R490) and SD3-SD1 (R567-E407, R563-E482) could stabilize the SsaV_C_ nonamer (Fig. 4B and Fig. S6A), which is consistent with the electrostatic surface potential analysis above and with previous studies (26, 44, 45, 47). SctV_L_ has also been reported to be required for forming the SctV_C_ ring (45, 50). In each SsaV_C_ monomer, the hydrophobic pocket at the connection region between SD1 and SD3 is occupied by a hydrophobic peptide (M346-V347-P348-G349-A350) from the neighboring SsaV_L_, forming the hydrophobic interactions between two adjacent subunits (Fig. 4C and Fig. S6B). To verify the importance of these two different intermolecular interactions for the SsaV_C_ nonameric structure formation, we created two variants, SsaV_C_M4 (E407A, E482A, E488A, R490A) and SsaV_C_N4 (deletion of M346-V347-P348-G349), and tested the oligomerization ability of these two variants through SEC. The results showed that neither variant could oligomerize in solution (Fig. 4D), indicating that both intermolecular salt bridges and hydrophobic interactions are essential for SsaV_C_ nonamerization.

The intermolecular salt bridges are remarkably conserved in all reported SsaV_C_ structures, consistent with a general role in maintaining the SctV_C_ ring structure (26, 44, 45, 47). The hydrophobic pocket at the connection region of the SD1 and SD3 is also exhibited in SsaV_C_ homologous proteins InvA_C_, CdsV_C_, EscV_C_, MxiA_C_ and FlhA_C_ (Fig. 4E). However, the hydrophobicity of the pocket-nested peptide from the neighboring SctV_L_ exhibits great diversity in different homologous proteins (Fig. 4F). InvA_C_, EscV_C_ and FlhA_C_ show relative hydrophobic pockets at the connection region of the SD1 and SD3. However, the interaction peptide from InvA_L_ (VSTET) is very hydrophilic, and those from EscV_L_ (ISPGA) and FlhA_L_ (SLGME) are less hydrophobic than SsaV_L_, reducing the hydrophobic interactions between neighboring subunits. The different strengths of intermolecular hydrophobic interactions provide possible explanations for why InvA_C_ is unable to form the nonameric ring in solution (Fig. 1B and C) and why the ring structures of EscV_C_ and FlhA_C_ are disassembled in high-concentration salt buffer (32, 45). The relatively strong hydrophobic interactions via the unique amino acid sequence of SsaV_L_ and the conserved salt bridges between adjacent subunits of SsaV_C_ can promote formation of a high-order oligomer in different conditions, which may evolve to adapt to the unique environment of the SCV.

### The SsaV linker region in chimeric ISS exhibits variable conformations.

The structural and functional analysis above shows that the peptide between SsaV_L_ and SsaV_C_ plays an essential role in stabilizing the SsaV ring structure. The structural features of TM and linker regions of SctV were previously shown only using *in situ* cryo-ET method (12). To review more structural information of SsaV_L_ and InvA_TM_, we further processed the cryo-EM data of the chimeric ISS in two independent strategies (Fig. S7 in the supplemental material) via which linker conformational changes of SctV were observed.

**FIG 5:**
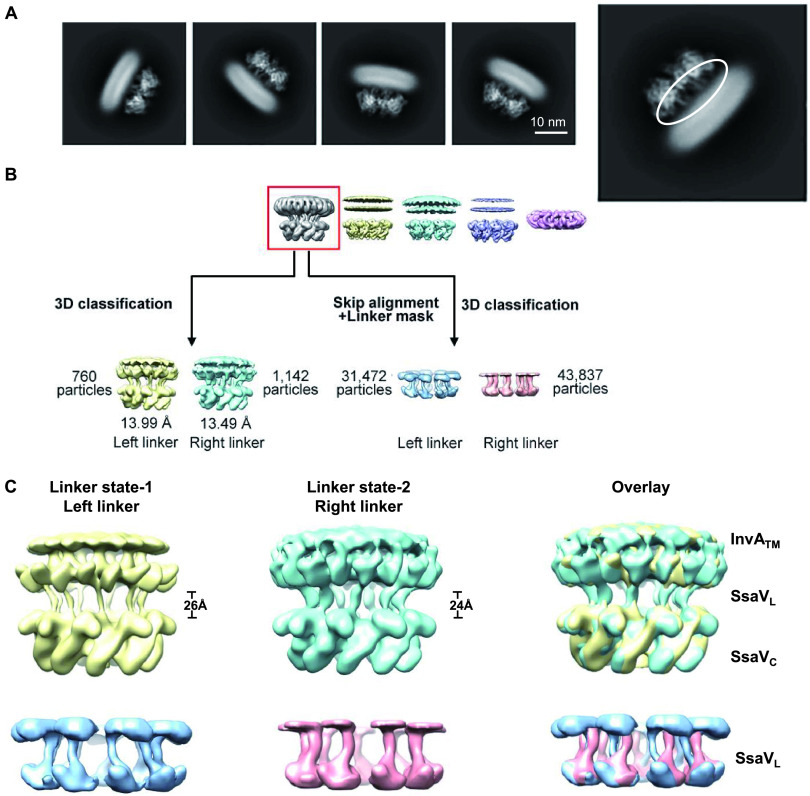
The linker region of the ISS exhibits variable conformations. (A) Representative 2D class averages of the chimeric ISS full-length protein. The linker region is clearly visible and highlighted with a white circle in the right panel. (B) Brief flowchart of EM data processing for the ISS linker region. A characteristic class featured with visible TM region and linker region (highlighted in red box) was obtained from 3D classifications (details in Materials and Methods and Fig. S7). Using 3D classification with this map as reference, the linker region was classified into variable conformations, with two major orientations, named left and right linkers (bottom-left panel). For independent validation of variable states of the linker region, a 3D classification by skipping alignment with a local mask in the linker region was performed and classes of the linker region in different orientations (left and right linker) were obtained (bottom-right panel). (C) Structural comparisons for linker regions of the ISS maps. Structural presentation and overlay comparison for left (colored in yellow) and right linker (colored in cyan) in whole maps of the ISS (top panel). Structural presentation and overlay comparison for left (colored in marine) and right linker (colored in pink) in maps of the ISS with a local mask in the linker regions (bottom panel). Notably, a few of features of TM region can be seen in the whole maps of the ISS.

In the side views of 2D classification, clear features of the SsaV_L_ region were captured between the SsaV_C_ ring structure and the blurry micelle of InvA TM domain (Fig. 5A). A characteristic class of ISS map featured with visible TM region and linker region (highlighted in the red box) was obtained from 3D classifications (details in Materials and Methods and Fig. S7). Using 3D classification with this map as the reference without a local mask, the linker region was classified into variable conformations, with two major orientations, featured as left and right linkers (Fig. 5B, bottom-left panel). For independent validation of variable states of the linker region, a 3D classification was performed by skipping alignment with a local mask in the linker region, and classes of the linker region in different orientations (left and right linker) were obtained (Fig. 5B, bottom-right panel). The linker region obtained from the two separate methods both exhibit two major states of SsaV_L_ with different orientations relative to SsaV_C_ in the fixed position (Fig. 5C and Fig. S7). The distance between InvA_TM_ and SsaV_C_ appears to vary in these two conformations at current resolution (Fig. 5C). The intriguing conformational changes of SsaV_L_ implies that SsaV might undergo a dynamic process during the substrate selection and secretion. However, more comprehensive and accurate information on the functional mechanism of SctV will require high-resolution SctV full-length structures in different conformations.

## DISCUSSION

As one of the engines of T3SS, SctV plays essential roles in effector selection and secretion. Due to the challenges in obtaining well-assembled SctV outside of the bacterial inner membrane, the structure and functional mechanism of SctV have been largely undefined. Through constructing the InvA_TM_-SsaV_C_ chimeric protein, we produced a homogenous high-order oligomeric SctV protein for cryo-EM structural study. However, we could only determine the high-resolution structure of nonameric SsaV_C_ and show a low-resolution map of SsaV_L_. A few of features of InvA_TM_ can also be observed (Fig. 5C and Fig. S7) but detailed structural information was lacked even after intense data processing. It might be resulted from the high flexibility of linker region, otherwise, InvA_TM_ might be intrinsically unstable or even unable to form the nonamer when it is extracted from the bacterial membrane, perhaps resulting from losing structural support from other partners of the T3SS. Further efforts may be needed to reconstitute SctV into the lipid membrane, perhaps with other potential interaction partners, to force SctV_TM_ to form the stable nonamer.

**FIG 6:**
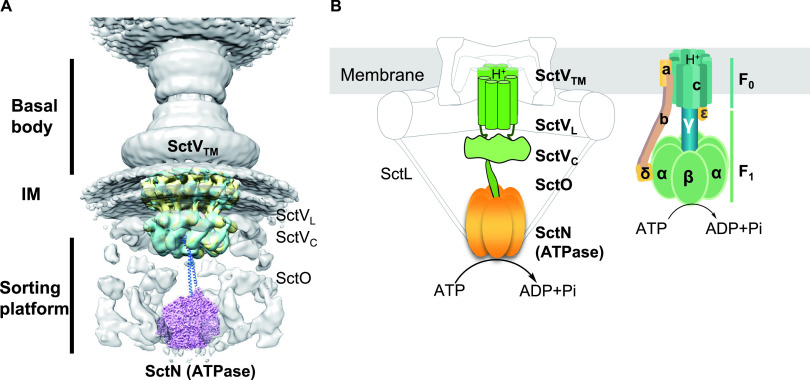
Model for putative mechanism in SctV coupling with ATPase complex. (A) Overview of the location of SctVON complex in the context of the Salmonella T3SS injectisome. SctV with two major states and the central stalk protein SctO (CdsO, marine) and the ATPase SctN (EscN, purple, PDB 6NJP) were docked into cryo-ET map of Salmonella Type III secretion injectisome map (EMD-8544). The CdsO structure was obtained by Phyre2 based on homology with YscO (PDB 4MH6). (B) Constructional, compositional and functional similarity between T3SS core engine system SctVON complex and F_0_F_1_-ATPase. Schematic diagrams of T3SS SctVON complex (left panel) and F_0_F_1_-ATPase (right panel). In F_0_F_1_-ATPase, the membrane-embedded c ring of F_0_ and hydrophilic ATPase F_1_ are two rotatory motors connected via the γ-subunit to translocate protons and generate a difference in potential by hydrolyzing ATP. In T3SS, the ATPase complex (SctN) is associated with the export gate (SctV) through interaction with the central stalk (SctO). The ATPase complex (SctN) generates energy by ATP hydrolysis in a rotary catalytic mechanism, which may drive effectors unfolding and secretion coupling with PMF generated by SctV in a cooperative manner.

It has been hypothesized that T3SS is energized by the ATPase-dependent ATP hydrolysis coupled with the proton-motive force (PMF) to secrete unfolded effectors, which is executed by the complex of export gate protein (SctV), center stalk protein (SctO), and ATPase (SctN) with a rotary catalytic mechanism on ATP hydrolysis consistent with the evolutionarily related F_0_F_1_-ATPase (25–28, 56–60). In F_0_F_1_-ATPase, the membrane-embedded c ring of F_0_ and hydrophilic ATPase F_1_ are two rotatory motors connected via the γ-subunit to translocate protons and generate a difference in potential by hydrolyzing ATP (56, 60). However, how the PMF and ATP hydrolysis coupling for the substrate secretion of T3SS is largely unknown. In our study, the 3D classification of SsaV_L_ shows two major orientations of SsaV_L_, implying that SsaV_L_ could be very dynamic during its functioning. The dynamic SctV_L_, SctV_C_, and SctO might form a bridge to coordinate the coupling between the SctV_TM_ (PMF) and SctN (ATP hydrolysis) to facilitate the substrate secretion of T3SS in an efficient manner (Fig. 6A). The dynamic conformation of SctV_L_ may also provide a structural explanation for the resent model for the action of FlhA in flagellar export that the FlhA_C_/SctV_C_ need to move to the FlhA_TM_/SctV_TM_ back and forth during the secretion cycle (49). Furthermore, considering the constructional, compositional and functional similarity between T3SS core engine system (SctVON) and F_0_F_1_-ATPase (Fig. 6B), as well as our observation of highly dynamic loop region of SctV in the full-length context, suggests an interesting hypothesis that T3SS may share a conserved rotary catalytic mechanism (SctV and SctN as two rotatory motors coupled via the SctO) with F_0_F_1_-ATPase to generate energy for protein secretion. However, this rotating model during SctV functioning is highly speculative. Further high-resolution full-length SctV structures and functional assays are imperative to fully dissect the molecular mechanism of how T3SS is energized for protein selection and secretion.

SctV_C_ was reported to involve substrate selection and secretion through the intramolecular cleft formed by SD2 and SD4 and the intermolecular cleft formed by two neighboring SD4s (13, 32). The SD2-SD4 cleft is dynamic and ranges from open to closed to selectively bind and release effector-chaperon pairs (13). The SD4-SD4 cleft has been shown to interact with central stalk protein SctO to facilitate the connection between SctV and ATPase SctN (26, 61). Due to the essential function of SctV in the T3SS secretion process, blockage of these two vital clefts of SctV should significantly diminish the function of T3SS and the virulence of the T3SS-employing bacterial pathogens (59). Therefore, SctV_C_ could be considered a potential novel target for developing anti-virulence drugs to some antibiotic resistant Gram-negative bacterial pathogens.

In this paper, we present near atomic-level insights into the assembly and functional mechanism of SctV_C_ and report variable states of SctV linker region. This study sheds light on important but heretofore poorly understood aspects of the remarkably complex biology of T3SS export gate protein SctV and thus has important implications for the development of therapeutic strategies targeting SctV_C_ to combat T3SS-mediated bacterial infection.

## MATERIALS AND METHODS

### Expression and purification of SsaV_C_ and InvA_TM_-SsaV_C_ chimera.

The DNA for SsaV_C_ (encoding residues 346–682) was cloned into pET15b (Novagen, Gibbstown, NJ) with a thrombin-cleavable His_6_ tag at the N-terminus. To generate the InvA_TM_-SsaV_C_ chimera, residues 316–685 of InvA were replaced by residues 326–682 of SsaV through Gibson assembly (62). All primers used in this study are listed in Table S2 in the supplemental material and all constructs were checked by DNA sequencing. An N-terminal Strep-tag and a SUMO protein in tandem were fused with InvA_TM_-SsaV_C_ chimera. Overexpression in Escherichia coli BL21 was induced overnight with 0.2 mM isopropyl-β-d-thiogalactopyranoside (IPTG) at 22°C when OD_600_ reached 0.8 (for InvA_TM_-SsaV_C_ chimera, OD_600_ = 1.2).

For purification of SsaV_C_, culture was harvested by centrifugation at 5,050 × *g* for 15 min at 4 °C and resuspended in lysis buffer containing 20 mM Tris-HCl (pH 8.0) and 150 mM NaCl and disrupted through a high-pressure homogenizer. After centrifugation at 17,000 × *g* for 50 min at 4 °C, the supernatant was applied into Ni-NTA resin (Qiagen, Hilden, Germany) and washed three times with 10 ml lysis buffer plus 20 mM imidazole. The protein was then eluted with lysis buffer with 300 mM imidazole, and further purified through an anion-exchange column (Hitrap Q, GE Healthcare, Fairfield, CT). Peak fractions were pooled and concentrated using 10 kDa cutoff Centricon filters (Millipore, Boston, MA) and subjected to size exclusion chromatography (Superdex 200 Increase 10/300 GL, GE Healthcare) in the lysis buffer. Finally, peak fractions were collected and concentrated to 5 mg/ml by 100 kDa cutoff Centricon filters for cryo-EM analysis.

To purify the InvA_TM_-SsaV_C_ chimera protein, 6 L of culture was collected, resuspended in lysis buffer, and disrupted through a high-pressure homogenizer. Insoluble fractions were removed by centrifugation at 20,000 × *g* for 20 min, while the supernatant was further ultracentrifuged at 150,000 × *g* for 1 h. The pellet (containing the membrane fraction) was resuspended in lysis buffer supplemented with 1% (wt/vol%) GDN and incubated at 4°C overnight. After centrifugation at 150,000 × *g* for 30 min, the supernatant was applied into Strep-Tactin Beads (Smart-Lifesciences, Changzhou, China) by gravity and washed with buffer W (lysis buffer plus 0.004% [wt/vol%] GDN). The target protein was eluted with buffer W after the SUMO tag was cleaved on the beads. The eluent was concentrated with a 100 kDa cutoff Centricon filter and further purified through size exclusion chromatography (Superose 6 Increase, GE Healthcare) in buffer W. SsaV and InvA were purified using the same procedure. For detergent screening, detergents were changed from membrane extraction to Superose 6 column in the purification. For cryo-EM analysis, peak fractions were concentrated to ∼10 mg/ml using 100 kDa cutoff Centricon filters.

### Cryo-EM data acquisition.

Aliquots of 4 µl concentrated samples were applied to glow-discharged holey carbon-coated grids (Quantifoil Au R1.2/1.3, 200 mesh, Beijing Zhongjingkeyi Technology, Beijing, China). Grids were blotted for 3.5 s at 8 °C with 100% humidity and frozen in liquid ethane using a Vitrobot Mark IV (Thermo Fisher Scientific, Waltham, MA). Grids were transferred to a Titan Krios (Thermo Fisher Scientific) operating at 300 kV and equipped with Gatan K3 Summit detector (Pleasanton, CA) and a GIF Quantum energy filter (slit width 20 eV). Micrographs were recorded in the super-resolution mode with a nominal magnification of 105,000x, resulting in a calibrated pixel size of 0.422 Å. Each stack of 32 frames was exposed for 2.13 s with an exposing time of 0.067 s per frame. The total dose was ∼ 50 e-/Å^2^ for each stack. AutoEMation (63) was used for the fully automated data collection. All 32 frames in each stack were aligned and summed using the whole-image motion correction program MotionCor2 (64) and binned to a pixel size of 0.8433 Å. The defocus value of each image was set to −0.8 μm to −1.5 μm and determined in cryoSPARC (65).

### EM data processing for SsaV_C_.

The data acquisition of SsaV_C_ is described as above Cryo-EM data acquisition section (named as data set 1). Out of 4,020 micrographs, 1,987,239 particles were automatically picked by cryoSPARC. After two rounds of 2D classification using cryoSPARC, a small subset of good particles was selected to generate the initial model; 584,301 good particles after 2D classification were used for 3D classification with C9 symmetry using cryoSPARC. Double-layer class (111,741 particles) and single-layer class (90,916 particles) were classified and further processed using Non-Uniform refinement, with D9 and C9 symmetry, respectively, resulting in double-layer map at 3.55 Å and single-layer map at 3.64 Å. A flowchart showing the data processing is shown in Fig. S1 in the supplemental material.

### EM data processing for InvA_TM_-SsaV_C_ chimeric protein (ISS).

#### (i) Data processing for SsaV_C_ of ISS.

The data acquisition of chimeric ISS is described as above Cryo-EM data acquisition section (named as data set 2). Out of 11,550 micrographs, 3,673,478 particles were automatically picked by cryoSPARC. After two rounds of 2D classification using cryoSPARC, a small subset of good particles was selected to generate the initial model: 1,225,081 good particles after 2D classification were used for 3D classification with C9 symmetry using cryoSPARC, and 734,284 good particles from the 3D classification were processed with further nonuniform refinement and local CTF refinement with C9 symmetry, resulting in the SsaV_C_ EM map with an averaged resolution at 2.11 Å. Features of the TM region and linker region of ISS are invisible in this map at current resolution. A flowchart showing the data processing is shown in Fig. S2 in the supplemental material.

#### (ii) Data processing for Initial model of InvA_TM_.

For checking the sample quality of the InvA_TM_-SsaV_C_ chimeric protein, 168 micrograph stacks were recorded using Talos Arctica (Thermo Fisher Scientific) at 200 kV equipped with a K2 detector (Pleasanton, CA), with motion correction using with MotionCor2 and CTF estimation using cryoSPARC, resulting in a calibrated pixel size of 1.17 Å. Out of 168 micrographs, 52,849 particles were automatically picked by cryoSPARC. After two rounds of 2D classification using RELION3.0 (66), a small subset of good particles was selected to generate the initial model; 44,442 good particles after 2D classification were used for 3D classification with C9 symmetry using RELION3.0. A representative InvA_TM_ map with featured TM region was obtained from 3D classification (Fig. S7A, red box, Model 1, 14,370 particles). A flowchart showing the data processing is shown in Fig. S7A.

#### (iii) Data processing for InvA_TM_ and SsaV_L_ without local mask.

For the InvA_TM_-SsaV_C_ chimeric protein described as above (data set 2), 375,338 particles in side views after 2D classifications were selected to process with 3D classification using Model 1 obtained above as the reference with C9 symmetry using RELION3.0, resulting in a full-length EM map class of InvA_TM_-SsaV_C_ chimeric protein with features of InvA_TM_, SsaV_L_, and SsaV_C_ (Fig. S7B, red box, Model 2, 28,922 particles). After two rounds of 3D classification, ISS EM maps with different linker states (Left’, Left, Right, Right’) were obtained. Representative EM maps (Left linker, 13.99 Å and Right linker, 13.49 Å) were obtained after refinement using RELION3.0. Reported resolutions were calculated on the basis of the FSC 0.143 criterion. A flowchart showing the data processing is shown in Fig. S7B in the supplemental material.

#### (iv) Data processing for SsaV_L_ with local mask.

For further estimating linker states of SsaV, 546,372 particles after 2D classification described above (Data set 2) were used to run a 3D classification by skipping alignment, using Model 2 as the reference, with a local mask in the linker region and C9 symmetry using RELION3.0. Two distinguished classes featured with left linker (31,427 particles) and right linker (43,387 particles) were obtained from the 3D classification. A flowchart showing the data processing is shown in Fig. S7C in the supplemental material.

### Model building and structure refinement.

The 2.11 Å reconstruction map was used for model building. The starting model of SsaV_C_ based on the structure of SsaV_C_ (PDB:7AWA) was manually built in Coot (67), followed by refinement against the corresponding maps in PHENIX (68) with secondary structure and geometry restraints. Statistics of 3D reconstruction and model refinement are summarized in Table S1 in the supplemental material. Structural figures were made using PyMOL v.2.3.2 (69) and UCSF ChimeraX v.1.1 (70). Analysis of sequence conservation was determined by the ConSurf server (71) according to sequence alignment using ClustalW (72). The pore diameter diagram calculated using the Hole program (73) in Coot. Phyre2 (74) was used to model the protein structures.

### DLS measurement.

DLS measurements were carried out using cuvette-based systems on a DynaPro NanoStar (WYATT, Santa Barbara, CA). Purified proteins were diluted to 0.5 mg/ml in lysis buffer containing 20 mM Tris-HCl (pH 8.0) and 150 mM NaCl. After centrifugation at 17,000 × *g* for 5 min, aliquots of 8 µl of samples were analyzed at 25 °C.
