# Supplementary material for: Structural and Functional Analysis of SsaV Cytoplasmic Domain and Variable Linker States in the Context of the InvA-SsaV Chimeric Protein
Source: Microbiol Spectr. 2021 Dec 1;9(3):e01251-21. doi: 10.1128/Spectrum.01251-21 (PMC8635156; doi:10.1128/Spectrum.01251-21)
Supplement: SUPPLEMENTAL FILE 1 — Supplemental material. Download SPECTRUM01251-21_Supp_1_seq10.pdf, PDF file, 1.7 MB [file spectrum01251-21_supp_1_seq10.pdf]

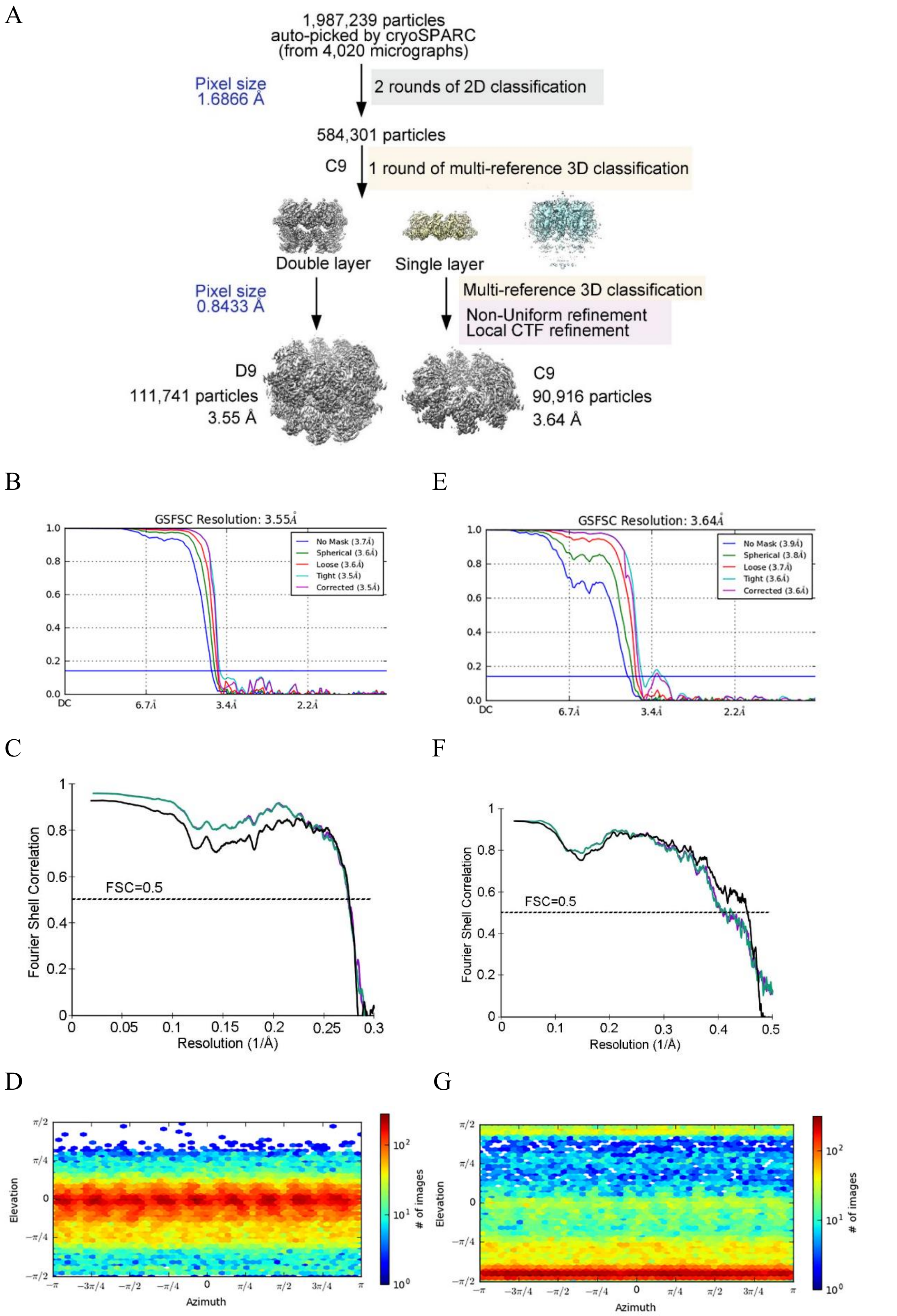

Figure S1. Cryo-EM analysis of SsaV$_C$. (A) Flowchart for EM data processing of SsaV$_C$. Double layer SsaV$_C$ with D9 symmetry and single layer SsaV$_C$ with C9 symmetry were reconstructed at a resolution of 3.55 Å and 3.64 Å, respectively. Details can be found in Methods. (B) Gold standard Fourier Shell Correlation (FSC) curves of

double layer $SsaV_C$ with D9 symmetry. (C) FSC curves of the refined double layer $SsaV_C$ PDB model versus the double layer $SsaV_C$ map that it is refined against (black); of the model refined against the first half map versus the same map (purple); and of the model refined against the first half map versus the second half map (green). The small difference between the purple and green curves indicates that the refinement of the atomic coordinates did not suffer from overfitting. (D) Angular distribution of particles used for reconstruction of double layer $SsaV_C$. (E-G) FSC curves and angular distribution of particles used for reconstruction of single layer $SsaV_C$ with C9 symmetry.

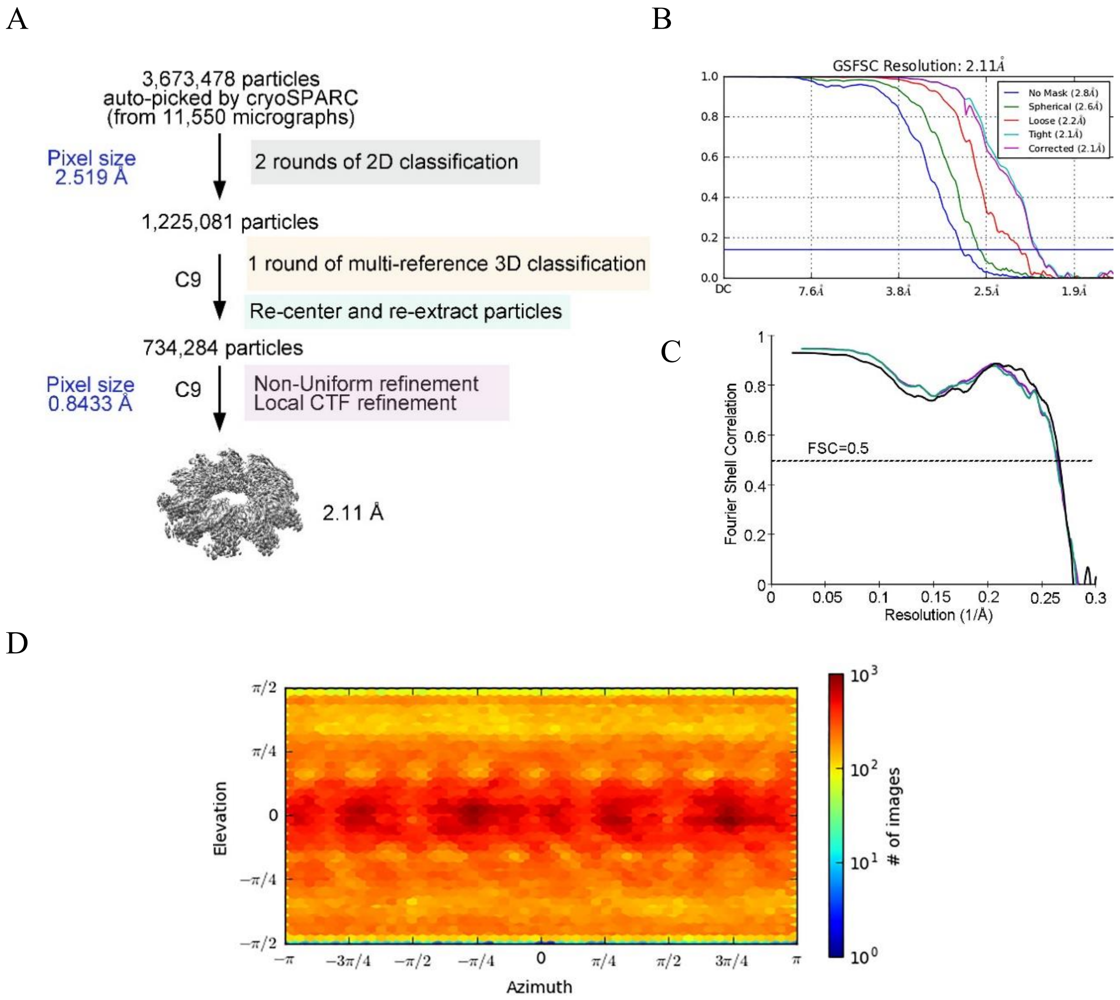

Figure S2. Cryo-EM analysis of the chimera full-length protein ISS. (A) Flowchart for EM data processing of ISS. A SsaV$_C$ map was reconstructed at 2.11 Å. Details can be found in Methods. (B) The gold-standard Fourier shell correlation (FSC) curves for the 3D reconstructions. (C) FSC curves of the refined SsaV$_C$ model versus the map that it is refined against (black); of the model refined against the first half map versus the same map (purple); and of the model refined against the first half map versus the second half map (green). The small difference between the purple and green curves indicates that the refinement of the atomic coordinates did not suffer from overfitting. (D) Particle angular distribution of the final reconstruction.

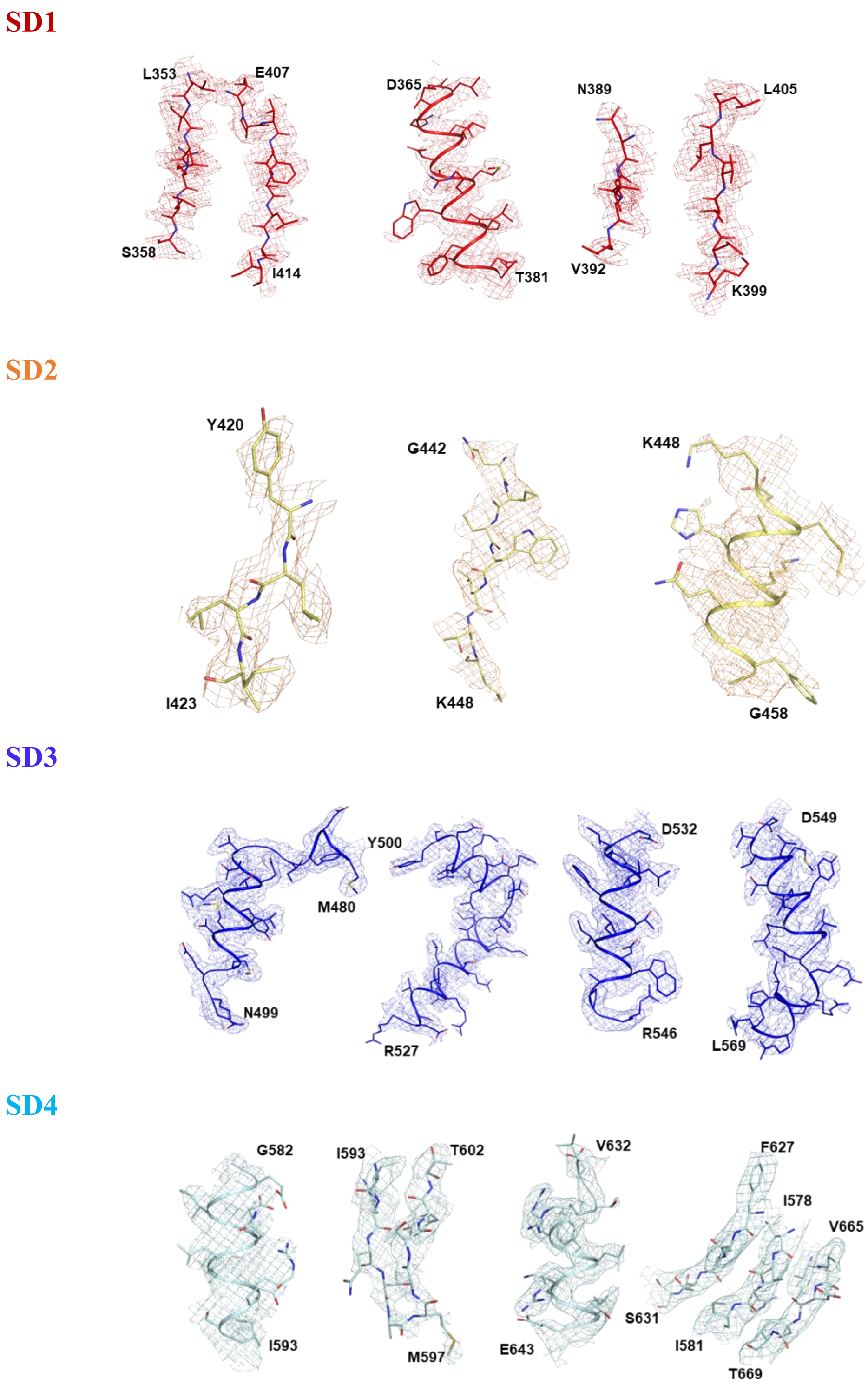

Figure S3. Representative EM densities in four subdomains of SsaV_C. EM maps for the indicated segments in SsaV_C, with SD1 domain colored in red, SD2 domain colored in orange, SD3 domain colored in blue, SD4 domain colored in green. The densities, contoured at 5-8 σ, were prepared in PyMOL.

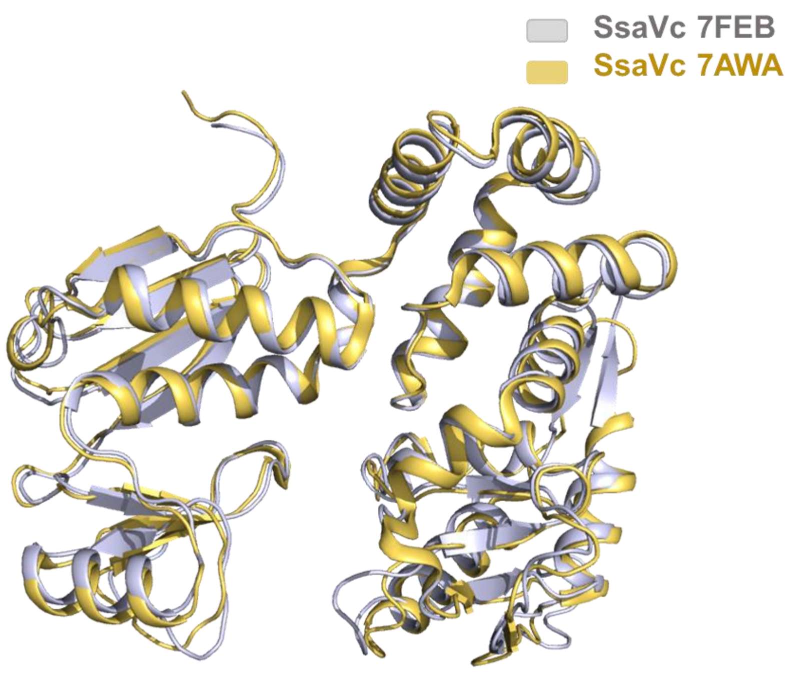

Figure S4. Superposition of the monomer of SsaVc (PDB: 7FEB, this study) and SsaVc (PDB: 7AWA). Structure of $SsaV_C$ (7FEB, this study) was superimposed to $SsaV_C$ (7AWA) in PyMOL. RMSD is 1.3598 Å over 312 residues between two structures.

A

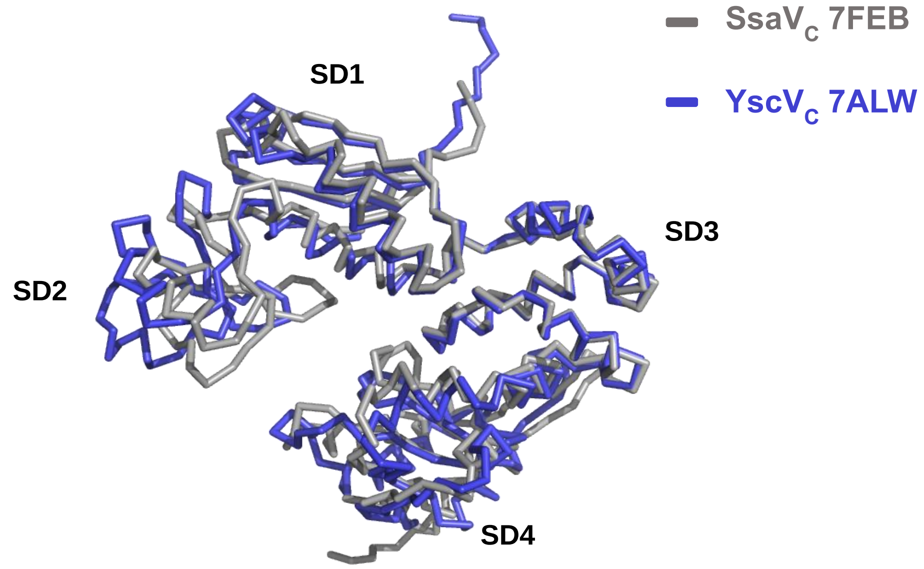

B

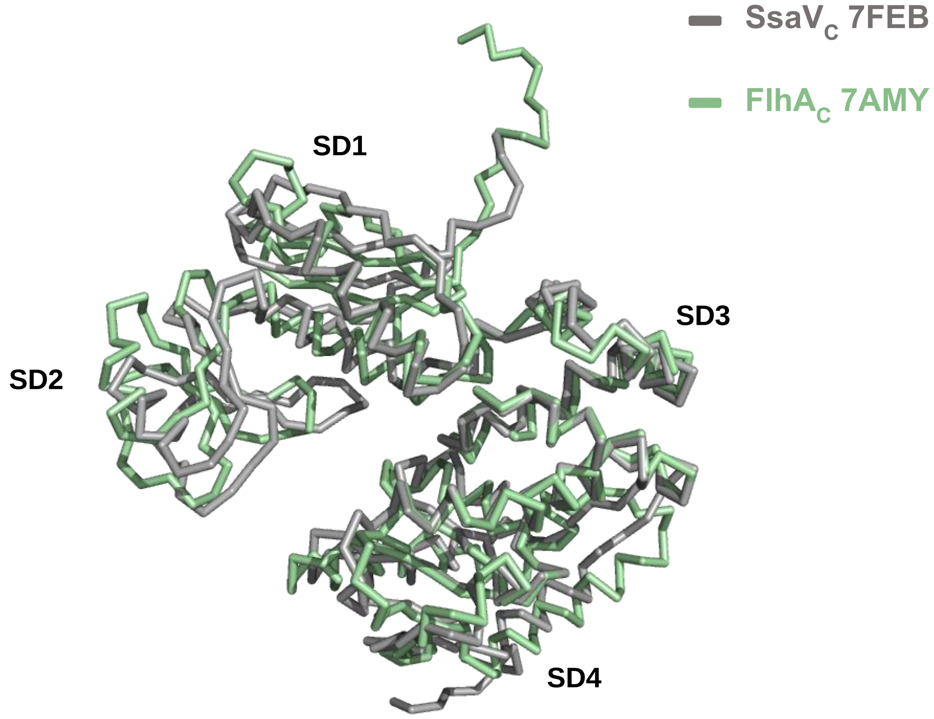

Figure S5. Superposition of the monomer of SsaV$_C$ (PDB: 7FEB, this study) and YscV$_C$ (PDB: 7ALW) and FlhA$_C$ (PDB: 7AMY). Overlay of SD3 of SsaV$_C$ monomer in gray with YscV$_C$ in blue and FlhA$_C$ in green. The SsaV$_C$ monomer is shown in a semi-open conformation. All these cytoplasmic domain structures are in the full-length context.

A

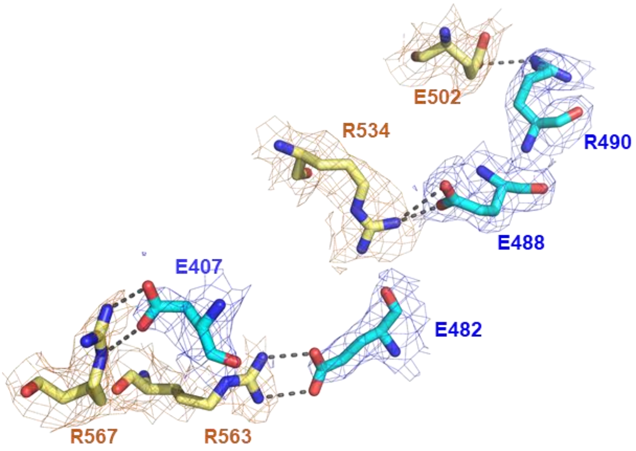

B

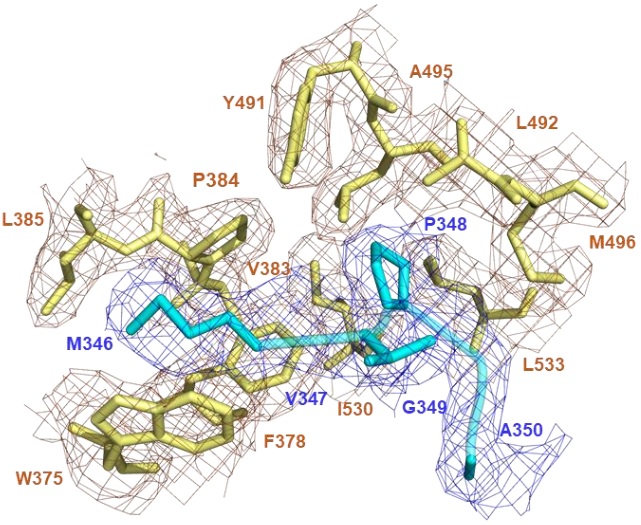

Figure S6. Detailed EM density presentation of interface between two adjacent monomers. (A) The EM densities for the residues from adjacent monomers (colored in yellow and cyan, respectively) that constitute salt bridges (related to Figure 4B). (B) The EM densities for residues involved in hydrophobic interactions, with residues from adjacent monomers shown as orange and blue mesh, respectively. EM densities are contoured at 5 σ and were prepared in PyMOL (related to Figure 4C).

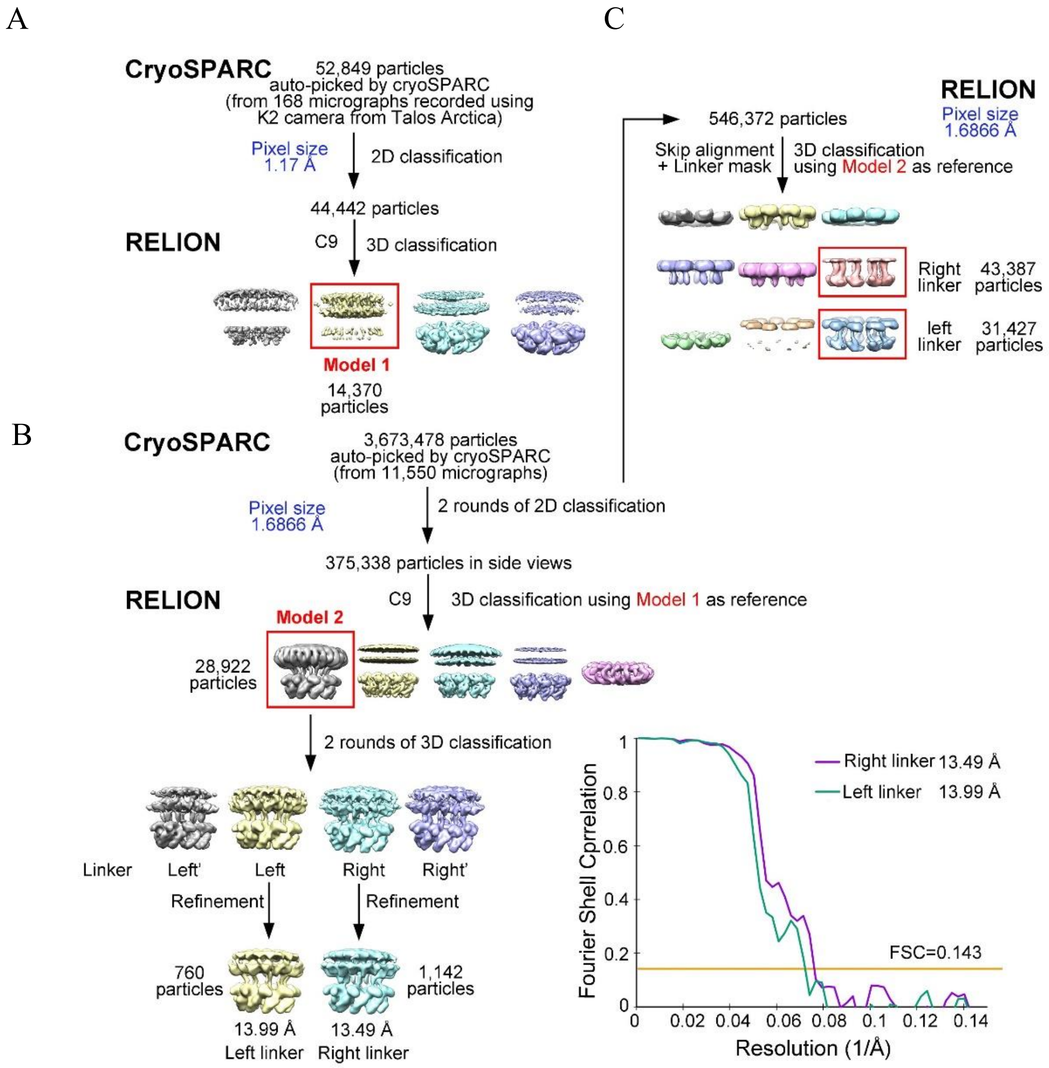

Figure S7. Flowchart for EM data processing of TM domain and linker region of chimera ISS. Details can be found in Methods. (A) EM data processing of ISS from micrographs recorded using Talos Arctica equipped K2 detector. A representative ISS_{TM} map (red box, Model 1) was obtained from 3D classification. (B) EM data processing of $InvA_{TM}$ and $SsaV_{L}$ from micrographs recorded using Titan Krios equipped with K3 detector and GIF. A full-length EM map of ISS including features of $InvA_{TM}$, $SsaV_{L}$, and $SsaV_{C}$ was obtained from 3D classification using Model 1 as a reference. After several rounds of 3D classification, ISS EM maps with different linker states (Left', Left, Right, Right') were obtained. Representative EM maps (Left linker, 13.99 Å and Right linker, 13.49 Å) were reconstructed after refinement using RELION3.0. Gold standard Fourier shell correlation (FSC) curves are shown in the bottom-right panel in B. (C) An alternative way for 3D classification of the ISS linker region by skipping alignment with a local mask in the linker region. $SsaV_{L}$ EM maps in state of Left linker and Right linker were also obtained.

Table S1. Cryo-EM data collection and refinement statistics.

**Dataset**	**SsaV$_C$ (ISS)**	**SsaV$_C$ C9**	**SsaV$_C$ D9**
**Data collection**			
EM equipment		Titan Krios (Thermo Fisher Scientific Inc.)	
Voltage (kV)		300	
Detector		Gatan K3	
Pixel size (Å)		0.8433	
Electron dose (e^-/Å^2)		50	
Defocus range (μm)		-0.8 to -1.5	
Number of movies	11,550	4,020	4,020
**Reconstruction**			
Software		cryoSPARC & RELION3.0	
Number of used particles	734,284	90,916	111,741
Symmetry	C9	C9	D9
Map sharpening B-factor (Å^2)	-79.7	-147.7	-176.8
Final resolution (Å)	2.11	3.64	3.55
**Model building and refinement**			
Software		PHENIX & COOT	
Protein residues	3033	3033	6006
R.m.s. deviations			
Bond length (Å)	0.005	0.006	0.002
Bond angle (°)	0.676	0.751	0.551
Ramachandran plot statistics (%)			
Favored	98.50	97.31	97.31
Allowed	1.5	2.69	2.69
Outlier	0	0	0
Clash score	14.22	15.76	12.14
MolProbity score	1.63	1.83	1.73
PDB code	7FEB	7FEC	7FED
EMDB code	EMD-31551	EMD-31552	EMD-31553

Table S2. Primers used in this study.

Name	Sequences (5'-3')	Notes
P1	CGCGCGGCAGCCATATGGTCTCTACAGAGACCGTAC	Used for amplify InvA$_C$355-685
P2	GCAGCCGGATCCTCGAGTTATATTGTTTTTATAACATTC	
P3	CCGCGCGGCAGCCATATGGTTCCCGGCGCATG	Used for amplify SsaV$_C$346-682
P4	GCAGCCGGATCCTCGAGTCATTCTTCATTGTCCGCC	
P5	CACAGAGAACAGATTGGTGGATCCATGCTGCTTTCTCTACTTAAC	Used for amplify InvA1-315
P6	CAGACTTTTTGCGGCGAATAAAATAGAAGAGTACGC	
P7	ATTCGCCGCAAAAAGTCTG	Used for amplify SsaV326-682
P8	GCAGCCGGATCTCAGTGTCATTCTTCATTGTCCGCC	
